# Early marriage and marital satisfaction among young married men in rural Uttar Pradesh, India

**DOI:** 10.1186/s13104-023-06271-9

**Published:** 2023-01-27

**Authors:** Shekhar Chauhan, T. V. Sekher

**Affiliations:** Department of Family and Generations, International Institute for Population Sciences, Mumbai, India

**Keywords:** ENRICH marital satisfaction scale, Education, Rural population, India

## Abstract

**Objective:**

Lack of reliable and valid scales of Indian origin prompt researchers to borrow the marital satisfaction scale developed in different settings. The lack of a reliable scale to understand marital satisfaction in India prompted us to examine the marital satisfaction among young married men using ENRICH Marital Satisfaction (EMS) Scale developed in the Western context. Assessing the reliability of the EMS scale on the rural population of Lalitpur and Shrawasti, Uttar Pradesh, India; this study examines the determinants of marital satisfaction among young married men.

**Results:**

Cronbach’s alpha coefficient of 0.936 confirms the high reliability of the EMS scale for the surveyed population in two districts of India. Men belonging to households with higher monthly income (OR- 3.33; 95% C.I. − 1.71–6.50) were more likely to be satisfied in their marriage than their counterparts. Similarly, fathers’, mothers’, and married men’s educational status were other important determinants of marital satisfaction. The study emphasizes the importance of family education as a strong predictor of marital satisfaction, and therefore policymakers may look into this aspect.

**Supplementary Information:**

The online version contains supplementary material available at 10.1186/s13104-023-06271-9.

## Introduction

Adult attachment theory states that people enter into a marital relationship to look for an attachment figure, a figure to whom they are emotionally close and in the presence of whom they feel secure [1]. The theory further implies that marriage is an institution where people derive fulfillment of their attachment needs and a sense of security. Therefore, marriage becomes a distinctive institute that effectively responds to people's needs [2]. The marital union acts as a source of individual happiness that helps them find fulfillment and meaning in their life [3]. People, therefore, enter marital unions seeking a satisfying and content way of life [4]. Marriage is the primary source of an individual’s happiness and meaning in life [5].

Marital satisfaction refers to an individual’s subjective evaluation of the marital relationship. The individual’s happiness in a marital union is possible only when their relationship is coherent and satisfactory [3]. Marital satisfaction is a cumbersome process that has over time been thought to be influenced by many factors, such as an individual’s education, socio-economic status, love, commitment, length of the marriage, sexual relations, presence of children, and division of labor [6].

An overview of the literature suggests that marriage and marital satisfaction studies have not received much attention in India than in Western countries [7]. The persistence of lower divorce rates in India has subsided the debate on marital satisfaction in the Indian population [8]. However, rising divorce trends in the recent period have given a push to studies related to marital satisfaction among couples in India [9, 10]. However, whatever limited research is available examining predictors of marital satisfaction among Indian couples, confirmed that financial security in the marriage, education status of the couple, and duration of marriage are some of the important predictors of marital satisfaction [11, 12]. The availability of limited research on early marriage and its impact on marital adjustment and marital satisfaction led to the concept of this study. This study aimed at examining marital satisfaction among men who were married early (below 21 years of age) in the Lalitpur and Shrawasti districts of Uttar Pradesh, India.

## Main text

### Data source

This study is based on the primary data collected in the Lalitpur and Shrawasti districts of Uttar Pradesh, India, from June to November 2019. Altogether 348 married young men (below the age of 25 years at the time of the survey) who married before attaining 21 years of age were personally interviewed. Twelve villages, six each from Lalitpur (Talbehat block) and Shrawasti (Hariharpur Rani block) districts, were selected for data collection. The selection of districts followed by block, villages and participants at the last was carried out using statistical sampling methods. The survey was conducted using structured schedules as provided through Additional files 3, 4.

### Outcome variable

Marital satisfaction was the outcome variable for this study. Marital satisfaction was measured through ENRICH Marital Satisfaction (EMS) Scale, a 15-item Likert scale. EMS scale was originally in English and it was translated in Hindi and again reverse translated in English to check its consistency. The Hindi version was also pilot tested to check its efficiency.

Each item on the scale was classified into five categories: varying from strongly disagree to strongly agree. Cronbach alpha reliability of the ENRICH marital satisfaction scale was 0.936. Value above 0.90 for Cronbach alpha is an excellent value [13–15]. Therefore, Cronbach alpha’s value indicates excellent reliability of the ENRICH marital satisfaction scale. Further, an index on a scale of three (low ‘0,’ medium ‘1,’ and high ‘2’) was created from 15 items of ENRICH marital satisfaction scale for detailed analysis (ordinal logistic regression analysis).

### Exposure variables

Exposure variables were divided into four groups; 1. Household factors; Caste [Scheduled Castes/Scheduled Tribes and Non-Scheduled Castes/Non-Scheduled Tribes (SC/ST and Non-SC/ST)], Religion (Hindu and Non-Hindu), Monthly Household Income (In Rs.) (Less than 5000, 5000–9999, 10000–19999, and 20000 and above), and Composition of Children (Equal number of sons and daughters, Daughters more, and Sons more/no daughter). 2. Father’s characteristics; Father’s education level (No education, up to the primary, up to secondary, and higher secondary and above), Age at which father got married (Below 21 years, and 21 and above years). 3. Mother’s characteristics; Mother’s education level (No education, up to the primary, up to secondary, and higher secondary and above) and Age at which mother got married (Below 18 years, and 18 and above years). 4. Married men’s characteristics; Education level (No education, up to the primary, up to secondary, and higher secondary, graduation and above).

### Statistical analysis

The study uses bivariate and ordinal logistic regression analysis. The ordinal logistic regression is used when the dependent variable is ordered with multiple categories. We have used ordered logistic regression to facilitate the interaction of dependent variable (having multiple ordered levels) with number of independent variables. The mathematical notation of the ordered logistic regression is given as follow:

$${\text{Logit }}\left[ {{\text{P}}\left( {{\text{Y}} \le {\text{j}}} \right)} \right] \, = \, \alpha_{{\text{j}}} {-} \, \sum \beta_{{\text{i}}} {\text{X}}_{{\text{i}}}$$

where j = 1, J-1 and i = 1,…, M

Here, j is the level of an ordered category with J levels, and i corresponds to independent variables.

### Ethical issues

The study proposal and survey questionnaires were approved by the Student Research Ethics Committee (SREC) of the International Institute for Population Sciences (IIPS), Mumbai, India. Informed consent was taken from each respondent. All the included participants were informed about the purpose of the study before data collection, and their identity was kept confidential.

## Results

The Cronbach alpha reliability of 0.936 suggests that the ENRICH marital satisfaction scale is reliable to measure marital satisfaction in the current context (Additional file 1: Table S1). Additional file 2: Table S2 depicts items in ENRICH Marital Satisfaction scale (15 items). EMS scale is subdivided into two categories: Marital Satisfaction (10 items) and Idealistic Distortion (5 items).

Table 1 shows the prevalence of marital satisfaction among married men in Lalitpur and Shrawasti as reported by them on the EMS scale. OMS 1 item examines the understanding between the couple, and around one-fifth (18.7%) of the men recorded their response as strongly disagree in OMS 1 item. About one-fourth (23.6%) of men recorded their response as strongly agree on OMS 13 item, stating ‘I have never regretted my relationship with my partner, not even for a moment.’

**Table 1:** Prevalence of (EMS, self-reported) level of marital satisfaction by men who were married below the legal age in Lalitpur and Shrawasti (N = 348)

EMS Scale items	Strongly disagree	Moderately disagree	Neither agree nor disagree	Moderately agree	Strongly agree
OMS 1	18.7	14.1	2.6	36.2	28.4
OMS 2	27	38.8	5.7	11.5	17
OMS 3	15.2	14.1	6.3	32.5	31.9
OMS 4	16.4	12.1	5.7	28.2	37.6
OMS 5	31.3	34.5	3.4	13.3	17.5
OMS 6	18.4	14.4	6.9	33	27.3
OMS 7	18.4	14.7	3.2	31.9	31.8
OMS 8	33.1	25.3	3.4	17.2	21
OMS 9	54.3	11.2	5.2	8.9	20.4
OMS 10	14.9	15.5	6.3	33.1	30.2
OMS 11	16.1	8.9	6.9	26.1	42
OMS 12	24.4	27.2	5.9	20.5	22
OMS 13	23.6	24.7	10.6	15.8	25.3
OMS 14	38.5	31.3	6.9	9.5	13.8
OMS 15	11.3	12.9	8	32.5	35.3

Table 2 depicts the marital satisfaction among married men in Lalitpur and Shrawasti by various background characteristics. Marital satisfaction among married men was significantly associated with the household’s monthly income, mother, father, and married men's education level. Around 28 percent of the men whose fathers were uneducated reported high marital satisfaction. More than two-fifths (45.7%) of the men whose fathers had graduation and above education reported high marital satisfaction. Similarly, around half of the uneducated men (50%) reported a low marital satisfaction level, whereas only 8.5 percent of men with graduation and above education reported low marital satisfaction. Almost one-fourth (25%) of the uneducated men reported high marital satisfaction, whereas almost half of the men (48.9%) with graduation and above education reported high marital satisfaction.

**Table 2:** Marital satisfaction level among married men by various background characteristics in Lalitpur and Shrawasti

Covariates	Low	Medium	High	N	Chi-square
Household’s characteristics					
Caste					
Non-scheduled caste/scheduled tribe	32.3	32.3	35.4	279	χ^2^ = 2.994 p < 0.224
Scheduled caste/scheduled tribe	39.1	36.2	24.6	69	
Religion					
Hindu	30.9	34.2	34.9	278	χ^2^ = 4.503 p < 0.105
Non-hindu	44.3	28.6	27.1	70	
Household income(monthly)in rupees					
Less than 5000	41.5	35.4	23.2	82	χ^2^ = 25.024 p < 0.000
5000–9999	33.1	40.4	26.5	151	
10000–19999	33.9	21	45.2	62	
20000 and above	22.6	22.6	54.7	53	
Father’s characteristics					
Father’s educational level					
No education	44	28	28	193	χ^2^ = 25.509 p < 0.000
Primary	28.6	33.3	38.1	63	
Secondary	19.3	42.1	38.6	57	
Higher Secondary and above	8.6	45.7	45.7	35	
Age at which father got married					
Below 21 years	33.2	34.6	32.2	295	χ^2^ = 2.207 p < 0.332
21 and above 21 years	35.8	24.5	39.6	53	
Mother’s characteristics					
Mother’s educational level					
No education	39.8	30.6	29.6	174	χ^2^ = 15.762 p < 0.015
Primary	23.8	38.1	38.1	216	
Secondary	13	52.2	34.8	84	
Higher secondary and above	12	20	68	23	
Age at which mother got married					
Below 18 years	33.4	34.5	32.1	293	χ^2^ = 2.027 p < 0.363
18 and above 18 years	34.5	25.5	40	55	
Married men’s characteristics					
Educational level					
No education	50	25	25	80	χ^2^ = 37.111 p < 0.000
Primary	53.5	30.2	16.3	43	
Secondary	33	33	34	100	
Higher secondary	21.8	37.2	41	78	
Graduation and above	8.5	42.6	48.9	47	

Table 3 shows the proportional odds model results estimating marital satisfaction among married men. Results found that men who belonged to Non-Scheduled Caste/Scheduled Tribe were 1.48 times more likely to report higher marital satisfaction than their counterparts. Results further noted that household income significantly contributed to marital happiness among married men. Those who belonged to households with 20000 and above monthly income were 3.33 times more likely to report marital happiness than their counterparts. Furthermore, fathers, mothers, and men’s education were significant predictors of marital satisfaction. Men whose fathers had higher secondary and above schooling were 3.19 times more likely to report marital satisfaction than those whose fathers had no education. Furthermore, men whose mothers had higher secondary and above schooling were 1.89 times more likely to report marital satisfaction than mothers with no education. Married men’s education was the biggest significant predictor of marital satisfaction. Men who had graduation and above education were 4.47 times more likely to report marital satisfaction than those men who were uneducated.

**Table 3:** Proportional odds model estimates for married men by their level of marital satisfaction after marriage among surveyed population

Covariates	OR	Standard error	C.I
Household’s characteristics			
Caste			
Scheduled caste/scheduled tribe®			
Non-Scheduled caste/scheduled tribe	1.48^a^	0.364	(0.19–2.39)
Religion			
Hindu®			
Non-Hindu	0.61	0.153	(0.37–1)
Household income(monthly)in rupees			
Less than 5000®			
5000–9999	1.30	0.327	(0.80–2.13)
10000–19999	2^b^	0.640	(1.06–3.75)
20000 and above	3.33^c^	1.13	(1.71–6.50)
Father’s characteristics			
Father’s educational level			
No education®			
Primary	1.82^b^	0.493	(1.07–3.09)
Secondary	2.21^c^	0.611	(1.29–3.80)
Higher secondary and above	3.19^c^	1.07	(1.65–6.15)
Age at which father got married			
Below 21 years®			
21 years and above	1.11	0.315	(0.64–1.94)
Mother’s characteristics			
Mother’s educational level			
No education®			
Primary	1.72	0.407	(1.08–2.74)
Secondary	1.94^a^	0.745	(0.91–4.12)
Higher secondary and above	1.89^a^	0.775	(0.84–4.22)
Age at which mother got married			
Below 18 years®			
18 years and above	1.17	0.324	(0.68–2.01)
Married Men’s characteristics			
Educational level			
No education®			
Primary	0.766	0.278	(0.37–1.56)
Secondary	1.90^b^	0.543	(1.08–3.32)
Higher secondary	2.87^c^	0.867	(1.58–5.18)
Graduation and above	4.47^c^	1.55	(2.25–8.84)

^a^,^b^, and ^c^ denote alpha value significance at 99 percent, 95 percent, and 90 percent confidenc interval. a = 0.01; b = 0.05; c = 0.1

® reference category, *C.I*. confidence interval

## Discussion

This article discusses marital satisfaction among early married males [16] believes that measures of marital quality developed in Western contexts should not be unthinkingly applied to non-Western contexts as some of the aspects may not be relevant in the non-Western contexts [16]. However, the high reliability of ENRICH marital satisfaction scale confirms the applicability in the context of the current study. The results are important in the Indian context, given the limited literature in the related field. The results found that men from Non-Scheduled Castes and households having a higher monthly income were more likely to be satisfied in their marriage than their counterparts. Furthermore, the parents' education status and the educational status of the men played a pivotal role in deciding marital satisfaction among men. The literature is scarce on marital satisfaction among men in the Indian context, limiting our understanding of the issue and restricting comparing the study findings with previous findings.

However, not in the Indian context, studies from different contexts noted a higher level of marital satisfaction among those with better financial status [17]. Imprudent financial behaviours lead to decreased family communications and diminishing quality of life, leading to poor marital satisfaction [18]. Also, mixed findings in this regard have been noted where individual happiness has strongly been correlated with having money [19], whereas other findings indicate that income and happiness are not associated [20]. A systematic review among Iranian men and women also noted economic factors as a determinant of marital satisfaction [21]. Allendorf, in her field-related research work, explicitly provided an explanation that could be attributed to higher marital satisfaction among men belonging to the richest class of household; she opines that- whenever she visited the members of wealthier families, she found them often sitting and chatting together in contrast to poor families who were often away in the fields for some subsistence activities [16]. This could make sense as wealthier people have more leisure time because of their economic status, thereby leading to better marital satisfaction.

In the Indian context, Caste remains a much debated and discussed topic [22, 23]. Caste is a social stratification system where one class of people is placed above others [24, 25]. It has been explicitly noted in the Indian context that certain Caste groups like SC/ST remain comparatively poor than Caste groups like Non-SC/ST [26]. It can be inferred that men who belonged to SC/ST households had poor marital satisfaction as they are from the poorer section of the society, as specified by Mishra & Bhardwaj (2020) [26]. To put the above inference in perspective, a study by Joshi et al. (2017) also noted higher marital communication among forward Caste (Non-SC/ST) [27]. In connection with several previous studies [28, 29], education status was another critical factor ascribed to marital satisfaction in this study population. Jyothi et al. (2020), in their primary study conducted in the rural set-up of Telangana, India, noted educational intervention as a precondition of improved marital satisfaction among couples [30].

## Conclusion

Given the scarcity of literature on marital satisfaction among young married men, this study is critical as it fills the much-awaited literature gap in the Indian context. Utilizing a Western scale to measure marital satisfaction in the Indian context, with the scale being reliable, is another contribution of this study. The study outlined specific determinants of marital satisfaction that can be explored further to bring some concrete policy suggestions later on.

## Limitations

The study findings are based on a small sample size and shall not be generalized to the national estimates. Furthermore, marital satisfaction is self-reported and some biasness towards responses cannot be ruled out.

## Supplementary Information

**Additional file 1**: **Table S1**. Correlation between items in ENRICH marital satisfaction scale (Inter-item reliability).

**Additional file 2**: **Table S2**. Items in ENRICH Marital Satisfaction Scale.

**Additional file 3**. Interview schedule for married men.

**Additional file 4**. Interview schedule for father.

## Data Availability

The datasets generated and/or analysed during the current study are available in the study itself. The survey questionnaires were designed specifically for this study and the same were approved by the ethical review board of the International Institute for Population Sciences, Mumbai, India.

## Abbreviations

EMS Scale: ENRICH marital satisfaction scale
SC: Scheduled Caste
ST: Scheduled Tribe
