# Supplementary material for: Early marriage and marital satisfaction among young married men in rural Uttar Pradesh, India
Source: BMC Res Notes. 2023 Jan 27;16:6. doi: 10.1186/s13104-023-06271-9 (PMC9881292; doi:10.1186/s13104-023-06271-9)
Supplement: Supplementary file 1 — Additional file 1: Table S1. Correlation between items in ENRICH marital satisfaction scale (Inter-item reliability). [file 13104_2023_6271_MOESM1_ESM.docx]

| **Additional file Table S1: Correlation between items in ENRICH marital satisfaction scale (Inter-item reliability)** | | | | | | | | | | | | | | | | |
| --- | --- | --- | --- | --- | --- | --- | --- | --- | --- | --- | --- | --- | --- | --- | --- | --- |
|  | oms01 | oms02 | oms03 | oms04 | oms05 | oms06 | oms07 | oms08 | oms09 | oms10 | oms11 | oms12 | oms13 | oms14 | oms15 | Cronbach’s alpha  Reliability |
| oms01 | 1 |  |  |  |  |  |  |  |  |  |  |  |  |  |  | 0.9469 |
| oms02 | 0.8244 | 1 |  |  |  |  |  |  |  |  |  |  |  |  |  | 0.9381 |
| oms03 | 0.8991 | 0.8603 | 1 |  |  |  |  |  |  |  |  |  |  |  |  | 0.9269 |
| oms04 | 0.8127 | 0.718 | 0.8105 | 1 |  |  |  |  |  |  |  |  |  |  |  | 0.9384 |
| oms05 | 0.7945 | 0.7933 | 0.8015 | 0.7912 | 1 |  |  |  |  |  |  |  |  |  |  | 0.9383 |
| oms06 | 0.8931 | 0.8023 | 0.886 | 0.8455 | 0.8197 | 1 |  |  |  |  |  |  |  |  |  | 0.9266 |
| oms07 | 0.8833 | 0.8105 | 0.8758 | 0.8055 | 0.7707 | 0.8783 | 1 |  |  |  |  |  |  |  |  | 0.9373 |
| oms08 | 0.6365 | 0.5877 | 0.6357 | 0.5647 | 0.6088 | 0.6726 | 0.5813 | 1 |  |  |  |  |  |  |  | 0.9414 |
| oms09 | 0.7634 | 0.6807 | 0.7355 | 0.6799 | 0.6898 | 0.7674 | 0.7321 | 0.5068 | 1 |  |  |  |  |  |  | 0.959 |
| oms10 | 0.8253 | 0.7321 | 0.8318 | 0.7648 | 0.7402 | 0.8607 | 0.8114 | 0.6636 | 0.7483 | 1 |  |  |  |  |  | 0.9273 |
| oms11 | 0.7322 | 0.6877 | 0.7239 | 0.6459 | 0.6622 | 0.7207 | 0.7327 | 0.4907 | 0.6866 | 0.7144 | 1 |  |  |  |  | 0.9293 |
| oms12 | 0.5597 | 0.5071 | 0.5392 | 0.464 | 0.5078 | 0.5793 | 0.5404 | 0.4617 | 0.5418 | 0.6126 | 0.5599 | 1 |  |  |  | 0.921 |
| oms13 | 0.6226 | 0.5864 | 0.6366 | 0.5857 | 0.5567 | 0.6826 | 0.6054 | 0.4742 | 0.56 | 0.6784 | 0.5268 | 0.4835 | 1 |  |  | 0.931 |
| oms14 | 0.6686 | 0.6582 | 0.7219 | 0.5898 | 0.6378 | 0.6823 | 0.6716 | 0.4925 | 0.6682 | 0.7107 | 0.6593 | 0.5669 | 0.5206 | 1 |  | 0.9296 |
| oms15 | 0.7507 | 0.7524 | 0.7803 | 0.6932 | 0.6791 | 0.7661 | 0.7283 | 0.5917 | 0.7223 | 0.7741 | 0.6948 | 0.5693 | 0.6374 | 0.7409 | 1 | 0.9483 |
| Cronbach’s α coefficient: 0.936 | | | | | | | | | | | | | | | | |
