# Supplementary material for: Early marriage and marital satisfaction among young married men in rural Uttar Pradesh, India
Source: BMC Res Notes. 2023 Jan 27;16:6. doi: 10.1186/s13104-023-06271-9 (PMC9881292; doi:10.1186/s13104-023-06271-9)
Supplement: Supplementary file 2 — Additional file 2: Table S2. Items in ENRICH Marital Satisfaction Scale. [file 13104_2023_6271_MOESM2_ESM.docx]

| **Additional file Table S2: Items in ENRICH Marital Satisfaction Scale** | | |
| --- | --- | --- |
| **Coding** | **Marital Satisfaction Scale** | **Sign** |
| OMS 2 | I am not pleased with the personality characteristics of my partner | -ve |
| OMS 3 | I am happy with how we handle role and responsibilities in our marriage | +ve |
| OMS 5 | I am not happy about our communication and feel my partner does not understand me. | -ve |
| OMS 7 | I am very happy about how we make decisions and resolve conflicts. | +ve |
| OMS 8 | I am unhappy about our financial position and the way we make financial decisions. | -ve |
| OMS 10 | I am very happy with how we manage our leisure activities and the time we spend together | +ve |
| OMS 11 | I am very pleased about how we express affection and relate sexually | +ve |
| OMS 12 | I am not satisfied with the way we each handle our responsibilities as parents | -ve |
| OMS 14 | I am dissatisfied about our relationship with my parents, in-laws, and/or friends. | -ve |
| OMS 15 | I feel very good about how we each practice our religious beliefs and values | +ve |
|  | **Idealistic Distortion Scale** | |
| OMS 1 | My partner and I understand each other perfectly | +ve |
| OMS 4 | My partner completely understands and sympathizes with my every mood | +ve |
| OMS 6 | Our relationship is a perfect success. | +ve |
| OMS 9 | I have some needs that are not being met by our relationships. | -ve |
| OMS 13 | I have never regretted my relationship with my partner, not even for a moment. | +ve |
| Average inter-item reliability : 0.691 & Cronbach’s alpha reliability: 0.936 | | |
