# Supplementary material for: Early marriage and marital satisfaction among young married men in rural Uttar Pradesh, India
Source: BMC Res Notes. 2023 Jan 27;16:6. doi: 10.1186/s13104-023-06271-9 (PMC9881292; doi:10.1186/s13104-023-06271-9)
Supplement: Supplementary file 3 — Additional file 3. Interview schedule for married men. [file 13104_2023_6271_MOESM3_ESM.docx]

**Child Grooms: A Study of Early Marriage and its Repercussions on Young Men in Rural Uttar Pradesh**

SCHEDULE NO:

CONFIDENTIAL

For Research Purpose Only


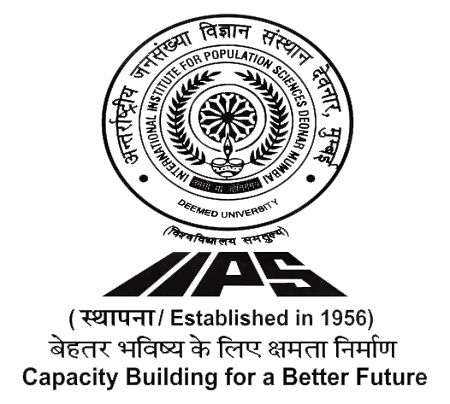


**Interview Schedule for Married Man**

| **IDENTIFICATION** | |
| --- | --- |
| DISTRICT: | VILLAGE: |
| TEHSIL: | NAME OF RESPONDENT: |
| DATE: |  |

| **Interview Status** | |
| --- | --- |
| Completed ………… 1 | Not at Home ………………. 3 |
| Incomplete ………… 2 | Refused ……………………. 4 |

**SECTION I: PERSONAL INFORMATION:**

| S. No. | Questions | Coding Categories | Skip/ Go to |
| --- | --- | --- | --- |
| 1. | What is your date of birth | Don’t Know  1A Day of Birth ………………... 98  1B Month of birth ……………… 98  1C Year of Birth ………………... 98 |  |
| 2. | How old were you on your last birthday? Compare and correct 1 and 2, if inconsistent | Age in completed years |  |
| 3. | What is your date of marriage | Don’t Know  3A Day of Marriage …………... 98  3B Month of Marriage ………… 98  3C Year of Marriage …………... 98 |  |
| 4. | How old were you when you got married? | Age in completed years |  |
| 5. | How many years you have completed in your married life? |  |  |
| 6. | Can you read and write? | Able to read only ………………… 1  Able to write only ………………... 2  Able to read and write ……………. 3  Cannot read or write ……………... 4 |  |
| 7. | What is the highest level of education that you have completed? | No education ……………………... 1  Primary …………………………... 2  Secondary ………………………... 3  Higher secondary ………………… 4  Graduation ……………………….. 5  Post-graduation or above ………… 6 |  |
| 8. | Years of schooling | 0 to 15 years |  |
| 9. | What is your current working status? | Currently working ………………... 1  Not working ……………………… 2 | Skip to Q 12 |
| 10. | What is your current occupation? | Government Job ………………….. 1  Private Job ……………………….. 2  Agricultural Labour (own land) ….. 3  Agricultural labour (Other’s land)…4  Animal Husbandry ……………….. 5  Any other (Specify) …………….. 96 |  |
| 11. | How much do you earn in a month? | In Rs. |  |
| 12. | Do you have children? | Yes …...…………………………... 1  Wife is pregnant now ……………. 2  No ………………………………... 3 | Skip to Q. 14 |
| 13. | If yes, Number of sons and daughters | Sons  Daughters |  |
|  | **Mass Media Exposure** | | |
| 14. | Do you read a newspaper or magazine? | Almost every day ………………… 1  At least once a week ……………... 2  Not at all …………………………. 3 |  |
| 15. | Do you listen to radio? | Almost every day ………………… 1  At least once a week ……………... 2  Not at all …………………………. 3 |  |
| 16. | Do you watch television? | Almost every day ………………… 1  At least once a week ……………... 2  Not at all …………………………. 3 |  |
| 17. | Do you go to movies/theatre? | At least once a week ……………... 1  At least once a month …………… 2  Not at all …………………………. 3 |  |

**SECTION II: REASONS FOR EARLY MARRIAGE AND MARRIAGE PRACTICES**

| S. No. | Questions | Coding Categories | Skip/ Go to |
| --- | --- | --- | --- |
| 18 | At what age did your parents start thinking about your marriage and started looking for alliances? | Below 10 years ………………...… 1  10-12 years ……………………….. 2  13-14 years ……………………….. 3  15- 16 years ………………………. 4  17- 18 years ………………………. 5  18-20 years ……………………….. 6  Don’t remember ………………… 98 |  |
| 19 | Why do you think they started looking for marriage proposals so early? | This is the custom in our community …………………………………… 1  My parents were poor ……………. 2  Most of my friends were getting married at that age …...…………… 3  Society started taunting my parents 4  My parents received a good marriage proposal …………………………... 5  They were offered a good dowry … 6  I stopped going to school ………… 7  I had sisters to be married after me 8  I was ready for marriage/ want to get married ………………. ………….. 9  Others (specify) …………………. 96 |  |
| 20 | How much time it took to arrange your marriage after parents started looking for it? | Within 6 months …………………. 1  6 months- 1 year …………………. 2  1- 2 years ………………………..... 3  2-3 years ………………………….. 4  More than 3 years ………………... 5 |  |
| 21 | Why you were married so early? | Everyone in our village do so ……. 1  After me, there were other siblings also to get married ………………... 2  Good dowry was offered …………. 3  Needed someone at home to manage household work …………………... 4  I was not responsible towards family responsibilities …………………… 5  I was not good at study and left the school ……………………………...6  I was ready for marriage/ want to get married ………………..………….. 7  Any other (specify) ……………... 96 | Skip  to Q26 |
| 22 | Did you want to get married at that time? marriage proposed for the first time | Yes ……………………………….. 1  No ………………………………... 2 | Skip to Q26 |
| 23 | If no, Why you did not want to get married at that time? | I was just a child …………………. 1  I wanted to study further …………. 2  I did not like the bride …………... 3  I did not feel safe to be married ….. 4  I do not have a job ………………. 5  Other (specify) ………………….. 96 |  |
| 24 | Did you try to convince your parents to delay the marriage? | Yes ……………………………….. 1  No ………………………………... 2 | Skip to  Q27 |
| 25 | Did they delay your marriage for few years? | Yes …...…………………………... 1  No ………………………………... 2 |  |
| 26 | If yes, why did you want to get married at that time? | It is normal to marry at that age ….. 1  I was excited to get married ……… 2  All my friends got married ……….. 3  I wanted to reduce the burden of my parents ……………………………. 4  I was fed up with the pressure upon me by society and family ……….... 5  Other (specify) ………………….. 96 |  |
| 27 | When did you come to know that your marriage was fixed? | Just before a week before marriage 1  Just before a month before marriage 2  1-6 months before marriage ……... 3  6-12 months before marriage……. 4  On the day of wedding …………… 5  Since childhood ………………….. 6 |  |
| 28 | How much time it took to have the marriage after fixing the marriage alliance? | Months ………………… |  |
| 29 | Were you excited/ happy about your marriage? | Yes …...…………………………... 1  No ………………………………... 2 |  |
| 30 | Did your parents ask your age preference of the girl for marriage? | Yes …...…………………………... 1  No ………………………………... 2 |  |
| 31 | Did your parents ask you for your bride preference for marriage? | Yes …...…………………………... 1  No ………………………………... 2 |  |
| 32 | Did you felt embarrassed to talk about your marriage related issues to your parents? | Yes …...…………………………... 1  No ………………………………... 2 |  |
| 33 | Did you get a chance to meet your spouse in person before marriage? | Yes …...…………………………... 1  No ………………………………... 2 |  |
| 34 | If no, When did you first saw your wife? | On the day of wedding …………… 1  After marriage ……………………. 2 |  |
| 35 | Did you get a chance to talk to your spouse before marriage? | Yes …...…………………………... 1  No ………………………………... 2 |  |
| 36 | Have you seen your spouse (photograph) before marriage? | Yes …...…………………………... 1  No ………………………………... 2 |  |
| 37 | How many proposals were seen/ screened before finalising your marriage? | First proposal …………………….. 1  1-3 proposals.……………………... 2  4-5 proposals ……………………... 3  More than 5 proposals …………… 4 | Skip to  Q 39 |
| 38 | What was the major reason for rejecting the proposals? | Family status of the girl was not as desired ……………………………. 1  Offered lesser dowry than expected 2  The girl was not good looking …… 3  The girl had affair ………………. 4  The girl was older than me ………. 5  The girl did not know how to carry out household work ……………… 6  The girl was more educated than me7  The girl was illiterate ……………. 8  Any other (Specify) …………….. 96 |  |
| 39 | If you had a choice, what type of marriage you would have preferred? | Marriage by choice ………………. 1  Arranged marriage ……………….. 2  Choice cum arranged …………….. 3  Doesn’t matter …………………… 4  Never want to get married …..…... 5 |  |
| 40 | Did you had anyone in mind for your marriage/ Did you like any girl? | Yes …...…………………………... 1  No ………………………………... 2 | Skip to  Q 49 |
| 41 | If yes, Did you tell that to your parents? | Yes …...…………………………... 1  No ………………………………... 2 | Skip to  Q 43 |
| 42 | If yes, did they agree? | Agreed ……………………………. 1  Were angry initially but agreed ….. 2  Did not agree at all ……………… 3 | Skip to  Q 44 |
| 43 | You did not tell your parents because | Scared ……………………………. 1  Not committed …………………… 2  Girl not ready to marry …………... 3  Other (specify) ………………….. 96 |  |
| 44 | Was she a person from your…? | School/college ……………………. 1  Friend’s circle …………………… 2  Neighbourhood …………………... 3  Relatives …………………………. 4  Other (specify) ………………….. 96 |  |
| 45 | Was she from your caste? | Yes …...…………………………... 1  No ………………………………... 2 |  |
| 47 | Since how long did you like this person? | Since childhood ………………… 1  Since few months ………………. 2  Few years ………………………. 3 |  |
| 48 | Do you regret now that you could not marry her? | Yes …...…………………………... 1  No ………………………………... 2 |  |
| 49 | Did you like the person whom your parents had chosen for you? | Yes …...………………………….. 1  No ………………………………... 2 |  |
| 50 | Do you think you deserve someone better? | Yes …...………………………….. 1  No ………………………………... 2 |  |
| 51 | Do you feel you were married too early? | Yes …...………………………….. 1  No ………………………………... 2 |  |
| 52 | In your society, is it common to get marry early? | Yes …...………………………….. 1  No ………………………………... 2 |  |
| 53 | Around at what age boys get married usually? | Age in years |  |
| 54 | At what age, girls get married usually? | Age in years |  |
| 55 | Did you know how much dowry was fixed for your marriage? | Knows approximately ……………. 1  Did not get dowry ……………….. 2  Don’t know …………………...… 98 | Skip to  Q 60  Q 57 |
| 56 | Please tell the approx. amount? | In Rs. |  |
| 57 | Did you use/utilise any dowry amount for your own purpose? | Yes …...………………………….. 1  No ………………………………... 2 |  |
| 58 | If yes, for what purpose? | To get a job ………………………. 1  For further education …………….. 2  To set a business …………………. 3  Any Other (Specify) ……………. 96 |  |
| 59 | In your opinion, early marriage of boys will help in getting more dowry? | Yes …...………………………….. 1  No ………………………………... 2 |  |
| 60 | In your opinion, arranging early marriage of boys will help in finding a better bride? | Yes …...………………………….. 1  No ………………………………... 2 |  |
| 61 | If you have to tell me about your participation in the mate selection, how would you rate it? | No participation ………………….. 1  Just informed, but no consent ...….. 2  Informed and consent taken ……… 3  Choice was given ………………… 4 |  |
| 62 | Did you witness any other child marriage? | Yes …...…………………………... 1  No ………………………………... 2 |  |
| 63 | Did you try to stop your marriage? | Yes …...………………………….. 1  No ………………………………... 2 |  |
| 64 | Who was mainly enforcing the marriage on you? | Father …………………………….. 1  Mother ……………………………. 2  Elder brother ……………………... 3  Elder sister ……………………….. 4  Other family members …………… 5  Relatives …………………………. 6  Neighbours ………………………. 7  Others …………………………… 96 |  |
| 65 | Was there any official attempt/effort to stop your child marriage? | Yes …...………………………….. 1  No ………………………………... 2 | Skip to  Q 67 |
| 66 | If yes, is it? | Sarpanch …………………………. 1  Panchayat ………………………… 2  District Magistrate ……………….. 3  Police …………………………….. 4  NGO ……………………………… 5  Community ………………………. 6  Any other (specify) ……………... 96 |  |
| 67 | Do you know the legal age at marriage of boys and girls in India? | Yes …...…………………………... 1  No ………………………………... 2 | Skip to  Q 68 |
| 67 A | For Boys | Age (in Years) |  |
| 67 B | For Girls | Age (in Years) |  |
| 68 | At what age, you would prefer to marry your son and daughter when they grow up? | Son (Age in Years)  Daughter (Age in Years) |  |
| 69 | Do you think legal age at marriage for boys should be increased or decreased? | Increased …………………………. 1  Decreased ………………………… 2  Keep it as it is now………………. 3 |  |
| 70 | Do you think legal age at marriage for girls should be increased or decreased? | Increased …………………………. 1  Decreased ………………………… 2  Keep as it is now …………………. 3 |  |

**SECTION III: EDUCATION AND EARLY MARRIAGE**

| S. No. | Questions | Coding Categories | Skip/ Go to |
| --- | --- | --- | --- |
| 71 | Did you like going to school? | Yes ……………………………….. 1  No ………………………………... 2 |  |
| 72 | How many years of schooling did you complete before marriage? |  |  |
| 73 | Did you have continuous schooling? | Yes ……………………………….. 1  No ………………………………... 2 | Skip to Q 77 |
| 74 | If no, what was the reason for gap in schooling? | Parents could not afford …………. 1  I had younger siblings to look after 2  I have to work to support family income …………………………… 3  I had to take care of my grandparents …………………………................ 4  I had to do household work ……… 5  School was far away from home … 6  I did not want to study …………… 7  My marriage was fixed …………... 8  I was harassed in the school ……... 9  Teachers were not good ………… 10  My friends had left going to school11  Other (specify) ………………….. 96 |  |
| 75 | How many years of gap you had in your schooling? | Years …………………. |  |
| 76 | Do you regret for failing to continue your education? | Yes ……………………………….. 1  No ………………………………... 2 |  |
| 77 | Are you still studying? | Yes ……………………………….. 1  No ………………………………... 2 | Skip to  Section IV |
| 78 | Who took the decision of leaving school/college | Myself …….……………………… 1  Father/ parents ………………….. 2  Any Other (Specify) ……………... 3 | Skip to Q 80 |
| 79 | Why you/father/parents decided to quit your schooling? | Parents could not afford …………. 1  I had younger siblings to look after 2  I have to work to add family income3  I had to take care of my grand parents …………………………................ 4  I had to do household work ……… 5  School was far away from home … 6  Not interested in studies .………… 7  My marriage was fixed …………... 8  I was harassed in the school ……... 9  Teachers were not good ………… 10  My friends had left going to school11  Other (specify) ………………….. 96 |  |
| 80 | Did you want to continue your education after marriage? | Yes ……………………………….. 1  No ………………………………... 2 |  |
| 81 | If given a chance, would you like to continue your education again? | Yes ……………………………….. 1  No ………………………………... 2 |  |
| 82 | How regular you were to school before you dropped out | Very Regular …………………….. 1  Regular …………………………... 2  Not Regular ……………………… 3 |  |
| 83 | Did you find it difficult to reach your school? | Yes ………………………………...1  No ………………………………... 2 |  |
| 84 | How far was the school from your home? | Very near ………………………… 1  Near ……………………………… 2  Very far …………………………... 3  Far ………………………………... 4 |  |
| 85 | How did you used to go to school? | Walking ………………………….. 1  Bicycle …………………………… 2  Rickshaw/ auto …………………... 3  School bus ……………………….. 4  Others …………………………... 96 |  |
| 86 | Which event occurred first in your life? | You left the school and then got married …………………………… 1  You marriage was fixed and then you discontinued school ……………... 2  You got married and then you left the school …………………………….. 3 | Skip to  Q 88  Q 89 |
| 87 | How long was the gap between you left the school and you got married? | Below 6 months ………………….. 1  Below 1 year ……………………... 2  More than a year …………………. 3  Below 2 years ……………………. 4  More than 2 years ………………... 5 |  |
| 88 | How long was the gap between your marriage was fixed and you left the school? | Below 6 months ………………….. 1  Below 1 year ……………………... 2  More than a year …………………. 3  Below 2 years ……………………. 4  More than 2 years ………………... 5 |  |
| 89 | How long was the gap between you got married and you left your school? | Below 6 months ………………….. 1  Below 1 year ……………………... 2  More than a year …………………. 3  Below 2 years ……………………. 4  More than 2 years ………………... 5 |  |
| 90 | Were you happy with your parent’s decision to leave school? | Yes ……………………………….. 1  No ………………………………... 2 |  |
| 91 | Is it common among your friends that they have to leave school for getting married? | Yes ……………………………….. 1  No ………………………………... 2 |  |
| 92 | Did you tried to convince your parents to continue school? | Yes ……………………………….. 1  No ………………………………... 2 |  |
| 93 | Did they allow you to continue studies? | Yes ……………………………….. 1  No ………………………………... 2 | Skip to  Q 95 |
| 94 | How many years of schooling you completed after marriage? | In Years |  |
| 95 | Do you still want to continue your education? | Yes ……………………………….. 1  No ………………………………... 2 |  |
| 96 | Do you ever felt that you should have completed your studies and then got married? | Yes ……………………………….. 1  No ………………………………... 2 |  |
| 97 | If you had studied, your life would have been better today? | Yes ……………………………….. 1  No ………………………………... 2 |  |
| 98 | Do you think boys and girls should be given equal opportunities to study? | Yes ……………………………….. 1  No ………………………………... 2 |  |
| 99 | In future, will you educate your daughter as much as you educate your son (if have or will have daughter and son)? | Yes ……………………………….. 1  No ………………………………... 2 |  |

**SECTION IV: JOB OPPORTUNITIES AND EARLY MARRIAGE:**

| S. No. | Questions | Coding Categories | Skip/ Go to |
| --- | --- | --- | --- |
|  | **For working married men** | |  |
| 100 | What happened first to you; your marriage or job | Marriage ………………………….. 1  Job ……………………………….. 2 | Skip to  Q105 |
| 101 | After your marriage, how long it took you to get a job? | (in Months) |  |
| 102 | Did you take up the job just because you were married by then? | Yes ……………………………….. 1  No ………………………………... 2 |  |
| 103 | If you could have delayed your marriage, you would have got a better opportunity to find a job? | Yes ……………………………….. 1  No ………………………………... 2 |  |
| 104 | Your friends who were married later than you, have got a better job than you? | Yes ……………………………….. 1  No ………………………………... 2 |  |
| 105 | Do you find it difficult to manage your household expenses with your current salary/income? | Yes ……………………………….. 1  No ………………………………... 2 | Skip to  Q107 |
| 106 | If yes, then what is the other sources of income for household expenses? | Agriculture ……………………….. 1  Parental support ………………….. 2  Parents-in-law support …………… 3  wife is working …………………... 4  Others (specify) …………………. 96 |  |
| 107 | Are you happy with your current job? | Yes ……………………………….. 1  No ………………………………... 2 | Skip to Q 110 |
| 108 | If no, are you looking for a new job? | Yes ……………………………….. 1  No ………………………………... 2 |  |
| 109 | Is it difficult at your place to find a good job? | Yes ……………………………….. 1  No ………………………………... 2 |  |
| 110 | Have you ever changed your job? | Yes ……………………………….. 1  No ………………………………... 2 | Skip to  Q 113 |
| 111 | If yes, how many times? | (in numbers) |  |
| 112 | What was the reason for changing your job? | Low salary in previous job ………. 1  Location was far from home ……... 2  Staff was not supporting …………. 3  Difficult to work …………………. 4  Health Reasons ………………….. 5  Any other (specify) ……………... 96 |  |
| 113 | Were you ready to take up a job, when you first got it? | Yes ……………….………………. 1  No ………………………..………. 2 |  |
| 114 | Do you think getting a job before marriage is important? | Yes ……………….………………. 1  No ………………………..………. 2 |  |
| 115 | Because you are married now, you cannot go far away in search for a better job? | Yes ……………………………….. 1  No ………………………………... 2 |  |
|  | **For unemployed married men** | |  |
| 116 | Are you interested in getting a job? | Yes …………………………….…. 1  No ………………………………... 2 | Skip to 127 |
| 117 | If no, then why you do not want to look for a job? | Father do not want me to take up a job ……………………………….. 1  I am single male child in my home 2  We have agricultural land to work 3  I do not want to go away from home in search of a job …….…………… 4  No burden on me to take a job …… 5  I am looking after family business 6  I have regular income from other sources ………………………….... 7  Any Other (Specify) ……………. 96 |  |
| 118 | Since how long you are looking for a job? | Before your marriage (In Years) …  After marriage (In Years) …….. |  |
| 119 | Do you think postponing your marriage would have increased your chances to get a job? | Yes ……………………………….. 1  No ………………………………... 2 |  |
| 120 | What is the most important reason that you are still unemployed? | Married early ……………………... 1 Less educated …………………….. 2  Uneducated ………………………. 3  Do not want to go far away ………. 4  No job opportunities available …… 5  Not qualified to get a job ………… 6  Parents want me at home ………… 7  Health Reasons …………………... 8  Any other (specify) ……………... 96 |  |
| 121 | Do you regret now for getting married early considering that you are not getting a job? | Yes ……………………………….. 1  No ………………………………... 2 |  |
| 122 | Because you are married now, you cannot go far away in search of a job? | Yes ……………………………….. 1  No ………………………………... 2 |  |
| 123 | Would you say that marrying early is the main reason for not able to get a job? | Yes ……………………………….. 1  No ………………………………... 2 |  |
| 124 | Did your parents help you in finding a job? | Yes ……………………………….. 1  No ………………………………... 2 |  |
| 125 | Did your parents-in-law help you in finding a job? | Yes ……………………………….. 1  No ………………………………... 2 |  |
| 126 | Did the dowry received was used to get a job for you? | Yes ……………………………….. 1  No ………………………………... 2 |  |
| 127 | Did your wife ever complained to you for not having a job? | Yes ……………………………….. 1  No ………………………………... 2 |  |
| 128 | Did your wife is interested to take up a job outside for her own? | Yes ……………………………….. 1  No ………………………………... 2 |  |
| 129 | Are you willing to allow your wife to take up a job? | Yes ……………………………….. 1  No ………………………………... 2 |  |
| 130 | Do you think, before marriage every man should have a job/ regular income? | Yes ……………………………….. 1  No ………………………………... 2 |  |

**SECTION V: FAMILY RESPONSIBILITIES:**

| S. No. | Questions | Coding Categories | Skip/ Go to |
| --- | --- | --- | --- |
| 131 | Do you find yourself too busy in managing your family responsibilities? | Yes ……………………………….. 1  No ………………………………... 2 |  |
| 132 | Do you think marrying early has overburdened you with the responsibilities? | Yes ……………………………….. 1  No ………………………………... 2 |  |
| 133 | Are you able to fulfil your family responsibilities properly? | Yes ……………………………….. 1  No ………………………………... 2 |  |
| 134 | Do you think that marrying later could have given you more time in understanding the family responsibilities? | Yes ……………………………….. 1  No ………………………………... 2 |  |
| 135 | Do you find yourself too young (in age) to take up the family responsibilities? | Yes ……………………………….. 1  No ………………………………... 2 |  |
| 136 | Do you think that your responsibilities have increased after your marriage? | Yes ……………………………….. 1  No ………………………………... 2 |  |
| 137 | Do you think that family responsibilities helped you in realising your maturity level? | Yes ……………………………….. 1  No ………………………………... 2 |  |
| 138 | Do you think that after marrying, you have become more responsible? | Yes ……………………………….. 1  No ………………………………... 2 |  |
| 139 | Are you able to decide what you will do each day? | Yes ……………………………….. 1  No ………………………………... 2 |  |
|  | How much do you agree with these statements? | |  |
| 140 | In our family, the father does not spend enough time with the children (If has children) | Strongly agree ……………………. 1  Moderately agree ………………… 2  Neither agree nor disagree ……….. 3  Moderately disagree ……………… 4  Strongly disagree ………………… 5 |  |
| 141 | Children seem to be a major source of problems in our relationship (if has children) | Strongly agree ……………………. 1  Moderately agree ………………… 2  Neither agree nor disagree ……….. 3  Moderately disagree ……………… 4  Strongly disagree ………………… 5 |  |
| 142 | Planning family activities is difficult because we misunderstand each other | Strongly agree ……………………. 1  Moderately agree ………………… 2  Neither agree nor disagree ……….. 3  Moderately disagree ……………… 4  Strongly disagree ………………… 5 |  |
| 143 | When you got married, were you mentally prepared to the role of a husband? | Yes ……………………………….. 1  No ………………………………... 2 |  |
| 144 | What was the age gap between you and your wife? | In Years |  |
| 145 | Did you find it difficult to adjust with your wife immediately after marriage/ | Yes ……………………………….. 1  No ………………………………... 2 |  |

**SECTION VI: EARLY MARRIAGE AND WELL-BEING:**

| S. No. | Questions | Coding Categories | Skip/ Go to |
| --- | --- | --- | --- |
|  | **A. Social well-being** | |  |
| 146 | Do you think that your life has been a failure? | Yes ……………………………….. 1  No ………………………………... 2 | Skip to  Q 148 |
| 147 | Is early marriage a cause for it? | Yes ……………………………….. 1  No ………………………………... 2 |  |
| 148 | Did your involvement with friends has reduced after your marriage? | Yes ……………………………….. 1  No ………………………………... 2 |  |
| 149 | Did you get time to spend with people now as you were having before marriage? | Yes ……………………………….. 1  No ………………………………... 2 |  |
| 150 | Are there enough people with whom you feel strongly connected? | Yes ……………………………….. 1  No ………………………………... 2 |  |
| 151 | Is your friend circle shrunken after your marriage? | Yes ………………………………...1  No………………………………… 2 |  |
| 154 | Do you now get the kind of support from your friends that you used to get before your marriage? | Not at all …………………………. 1  Not much ………………………… 2  Moderately ……………………….. 3  A great deal ………………………. 4  Completely ……………………….. 5 |  |
| 155 | Keeping in mind your situation before marriage, how well you are able to get around after marriage? | Not at all …………………………. 1  Not much ………………………… 2  Moderately ……………………….. 3  A great deal ………………………. 4  Completely ……………………….. 5 |  |
| 156 | Do you think that marrying early has curtailed your freedom to enjoy with friends | Yes ………………………………...1  No………………………………… 2 |  |
| 157 | Do you think that marrying early has done more harm than good to you? | Yes ………………………………...1  No………………………………… 2 |  |
|  | **B. Financial Well-being** | |  |
|  | How well does these statements describe you or your situation? | |  |
| 158 | I could handle a major unexpected expense | Describes me completely ………… 1  Describes me very well …………... 2  Describes me somewhat ………….. 3  Describes me very little ………….. 4  Does not describe me at all .……… 5 |  |
| 159 | I am securing my financial future | Describes me completely ………… 1  Describes me very well …………... 2  Describes me somewhat ………….. 3  Describes me very little ………….. 4  Does not describe me at all .……… 5 |  |
| 160 | Because of my financial situation, I feel like I will never have the things I want in life | Describes me completely ………… 1  Describes me very well …………... 2  Describes me somewhat ………….. 3  Describes me very little ………….. 4  Does not describe me at all .……… 5 |  |
| 161 | I am concerned that the money I have or will save won’t last | Describes me completely ………… 1  Describes me very well …………... 2  Describes me somewhat ………….. 3  Describes me very little ………….. 4  Does not describe me at all .……… 5 |  |
|  | How often does these statements apply to you? | |  |
| 162 | Giving a gift for a wedding, birthday or other occasion would put a strain on my finances for the month | Always …………………………… 1  Often ………………..……………. 2  Sometimes …...…………………… 3  Rarely …...………………………... 4  Never ……………………………... 5 |  |
| 163 | I have money left over at the end of the month | Always …………………………… 1  Often ………………..……………. 2  Sometimes …...…………………… 3  Rarely …...………………………... 4  Never ……………………………... 5 |  |
| 164 | How often do you run short of money for food or other regular expenses? | Always …………………………… 1  Often ………………..……………. 2  Sometimes …...…………………… 3  Rarely …...………………………... 4  Never ……………………………... 5 |  |
| 165 | How often do you save money so that you could cover major unexpected expenses or a fall in income? | Always …………………………… 1  Often ………………..……………. 2  Sometimes …...…………………… 3  Rarely …...………………………... 4  Never ……………………………... 5 |  |
| 166 | I always make sure I have money saved for bad times | Always …………………………… 1  Often ………………..……………. 2  Sometimes …...…………………… 3  Rarely …...………………………... 4  Never ……………………………... 5 |  |
| 167 | Do you think that marrying early has put an extra financial pressure on you? | Yes ……………………………….. 1  No ………………………………... 2 | Skip to  Q169 |
| 168 | How you cope up with that extra financial pressure? | Cutting your personal expenses ….. 1  By working extra hours ………….. 2  By not sending children to good school …………………………….. 3  By not utilising money on leisure activities ………………………….. 4  By borrowing money …………… 5  Any other (specify) ……………... 96 |  |
| 169 | Do you think that you will be able to secure your children’s future with your finances? | Yes ……………………………….. 1  No ………………………………... 2 |  |
| 170 | Do you put an extra effort to manage the household expenses? | Yes ……………………………….. 1  No ………………………………... 2 |  |

**SECTION VII: EARLY MARRIAGE AND SATISFACTION:**

| S. No. | Questions | Coding Categories | Skip/ Go to |
| --- | --- | --- | --- |
|  | **A. Marital satisfaction** | |  |
| 171 | Do you enjoy your wife's company? | Yes ……………………………….. 1  No ………………………………... 2 |  |
| 172 | Do you enjoy doing things together with your wife? | Yes ……………………………….. 1  No ………………………………... 2 |  |
| 173 | Being a husband at early age is satisfying? | Yes ……………………………….. 1  No ………………………………... 2 |  |
| 174 | Do you think people who married early are happier than those persons who marry late. | Yes ……………………………….. 1  No ………………………………... 2 |  |
| 175 | How satisfied are you with your marriage? | Very satisfied …………………….. 1  Satisfied ………………………….. 2  Neither satisfied nor dissatisfied …. 3  Dissatisfied ………………………. 4  Very dissatisfied …………………. 5 |  |
| 176 | How satisfied are you with your relationship with your wife? | Very satisfied …………………….. 1  Satisfied ………………………….. 2  Neither satisfied nor dissatisfied …. 3  Dissatisfied ………………………. 4  Very dissatisfied …………………. 5 |  |
|  | How much do you agree with these statements? | |  |
| 177 | I am well-adjusted with my wife. | Strongly agree ……………………. 1  Moderately agree ………………… 2  Neither agree nor disagree ……….. 3  Moderately disagree ……………… 4  Strongly disagree ………………… 5 |  |
| 178 | There are times when my wife does things that make me unhappy. | Strongly agree ……………………. 1  Moderately agree ………………… 2  Neither agree nor disagree ……….. 3  Moderately disagree ……………… 4  Strongly disagree ………………… 5 |  |
| 179 | Have you ever considered divorce, separation, or terminating your relationship? | Yes ……………………………….. 1  No ………………………………... 2 |  |
| 180 | Did your wife ever left for her parental home after a fight? | Yes ……………………………….. 1  No ………………………………... 2 |  |
| 181 | Looking back on some of the expectations you had before your marriage, have those changed now? | Yes ……………………………….. 1  No ………………………………... 2 |  |
|  | **B. Economic Satisfaction** | |  |
| 182 | Are you able to meet your daily expenses without any worries? | Yes ……………………………….. 1  No ………………………………... 2 |  |
| 183 | Do you struggle to meet your basic requirements? | Yes ……………………………….. 1  No ………………………………... 2 | Skip to  Q 185 |
| 184 | Do you think that your struggle to meet your basic requirements is due to your early marriage? | Yes ……………………………….. 1  No ………………………………... 2 |  |
|  | **C. Personal Satisfaction** | |  |
| 185 | Was that right time when you got married? | Yes ……………………………….. 1  No ………………………………... 2 |  |
| 186 | All things considered, how satisfied / happy are you with your life after marriage? | Very satisfied …………………….. 1  Satisfied ………………………….. 2  Not too satisfied…………………... 3 |  |
| 187 | Do you get enough time to communicate with your wife? | Yes ……………………………….. 1  No ………………………………... 2 |  |
| 188 | Are you happy with your life in general? | Yes ……………………………….. 1  No ………………………………... 2 |  |
| 189 | Are you happy with your family life? | Yes ……………………………….. 1  No ………………………………... 2 |  |
| 190 | Do you think that delaying your marriage could have made your life a bit happier? | Yes ……………………………….. 1  No ………………………………... 2 |  |
|  | How much do you agree with these statements? | |  |
| 191 | I am satisfied with my life | Strongly agree ……………………. 1  Moderately agree ………………… 2  Neither agree nor disagree ……….. 3  Moderately disagree ……………… 4  Strongly disagree ………………… 5 |  |
| 192 | If I could live my life over, I would change almost nothing as far as my marriage is concerned | Strongly agree ……………………. 1  Moderately agree ………………… 2  Neither agree nor disagree ……….. 3  Moderately disagree ……………… 4  Strongly disagree ………………… 5 |  |
| 193 | How would you rate your quality of life after marriage? | Very satisfied …………………….. 1 Satisfied ………………………….. 2 Neither satisfied nor dissatisfied …. 3 Dissatisfied ………………………. 4  Very dissatisfied …………............. 5 |  |
| 194 | To what extent, do you have the opportunity for leisure activities? | Not at all …………………………. 1  A little ……………………………. 2  Moderately ……………………….. 3  Mostly ……………………………. 4  Completely ……………………….. 5 |  |
| **D. OVERALL MARITAL SATISFACTION** | | | |
| 195 | My wife and I understand each other perfectly | Strongly agree ……………………. 1  Moderately agree ………………… 2  Neither agree nor disagree ……….. 3  Moderately disagree ……………… 4  Strongly disagree ………………… 5 |  |
| 196 | I am not pleased with the personality characteristics and personal habits of my wife. | Strongly agree ……………………. 1  Moderately agree ………………… 2  Neither agree nor disagree ……….. 3  Moderately disagree ……………… 4  Strongly disagree ………………… 5 |  |
| 197 | I am very happy with how we handle roles and responsibilities in our marriage. | Strongly agree ……………………. 1  Moderately agree ………………… 2  Neither agree nor disagree ……….. 3  Moderately disagree ……………… 4  Strongly disagree ………………… 5 |  |
| 198 | My wife completely understands and sympathises with my every mood. | Strongly agree ……………………. 1  Moderately agree ………………… 2  Neither agree nor disagree ……….. 3  Moderately disagree ……………… 4  Strongly disagree ………………… 5 |  |
| 199 | I am not happy about our communication and feel my wife does not understand me. | Strongly agree ……………………. 1  Moderately agree ………………… 2  Neither agree nor disagree ……….. 3  Moderately disagree ……………… 4  Strongly disagree ………………… 5 |  |
| 200 | Our relationship is a perfect success. | Strongly agree ……………………. 1  Moderately agree ………………… 2  Neither agree nor disagree ……….. 3  Moderately disagree ……………… 4  Strongly disagree ………………… 5 |  |
| 201 | I am very happy about how we make decisions and resolve conflicts | Strongly agree ……………………. 1  Moderately agree ………………… 2  Neither agree nor disagree ……….. 3  Moderately disagree ……………… 4  Strongly disagree ………………… 5 |  |
| 202 | I am unhappy about our financial position and the way we make financial decisions. | Strongly agree ……………………. 1  Moderately agree ………………… 2  Neither agree nor disagree ……….. 3  Moderately disagree ……………… 4  Strongly disagree ………………… 5 |  |
| 203 | I have some needs that are not met by our relationship. | Strongly agree ……………………. 1  Moderately agree ………………… 2  Neither agree nor disagree ……….. 3  Moderately disagree ……………… 4  Strongly disagree ………………… 5 |  |
| 204 | I am very happy with how we manage our leisure activities and the time we spend together. | Strongly agree ……………………. 1  Moderately agree ………………… 2  Neither agree nor disagree ……….. 3  Moderately disagree ……………… 4  Strongly disagree ………………… 5 |  |
| 205 | I am very pleased about how we express affection and relate sexually. | Strongly agree ……………………. 1  Moderately agree ………………… 2  Neither agree nor disagree ……….. 3  Moderately disagree ……………… 4  Strongly disagree ………………… 5 |  |
| 206 | I am not satisfied with the way we each handle our responsibilities as parents  **(NOTE: Ask only if the respondent has children)** | Strongly agree ……………………. 1  Moderately agree ………………… 2  Neither agree nor disagree ……….. 3  Moderately disagree ……………… 4  Strongly disagree ………………… 5 |  |
| 207 | I have never regretted my relationship with my wife, not even for a moment. | Strongly agree ……………………. 1  Moderately agree ………………… 2  Neither agree nor disagree ……….. 3  Moderately disagree ……………… 4  Strongly disagree ………………… 5 |  |
| 208 | I am dissatisfied about our relationship with my parents, in-laws, or friends. | Strongly agree ……………………. 1  Moderately agree ………………… 2  Neither agree nor disagree ……….. 3  Moderately disagree ……………… 4  Strongly disagree ………………… 5 |  |
| 209 | I feel good about how we each practice our religious beliefs and values. | Strongly agree ……………………. 1  Moderately agree ………………… 2  Neither agree nor disagree ……….. 3  Moderately disagree ……………… 4  Strongly disagree ………………… 5 |  |

**SECTION VIII: IMMEDIATE PROBLEMS AFTER MARRIAGE:**

| S. No. | Questions | Coding Categories | Skip/ Go to |
| --- | --- | --- | --- |
| Now, I am going to ask you some questions related to the time when you were married. So, try to give response keeping in mind the situation when you were newly married. | | | |
| 210 | Did you find it difficult to get along with your wife after your marriage? | Yes ……………………………….. 1  No ………………………………... 2 |  |
| 211 | Did you feel burden of taking care of your wife? | Yes ……………………………….. 1  No ………………………………... 2 |  |
| 212 | Did you find it difficult to take out time for your friends? | Yes ……………………………….. 1  No ………………………………... 2 |  |
| 213 | Did any of your friends ever tease you for getting married early? | Yes ……………………………….. 1  No ………………………………... 2 |  |
| 214 | Did you feel an extra responsibility after your marriage? | Yes ……………………………….. 1  No ………………………………... 2 |  |
| 215 | Did you feel economic pressure after your marriage? | Yes ……………………………….. 1  No ………………………………... 2 |  |
| 216 | Were you capable enough to manage your finances after marriage? | Yes ……………………………….. 1  No ………………………………... 2 |  |
| 217 | Were you given a comfortable space at your home after your marriage? | Yes ……………………………….. 1  No ………………………………... 2 |  |
|  | **LIFESTYLE RELATED QUESTIONS:** | |  |
| 218 | Did you find a change in your social connectivity after your marriage? | Yes ……………………………….. 1  No ………………………………... 2 |  |
| 219 | Did you notice that after your marriage, you were not able to meet your friends as frequently as you used to meet them before marriage? | Yes ……………………………….. 1  No …...…………………………… 2 |  |
| 220 | Did you say that due to marriage your leisure time is cut off? | Yes ……………………………….. 1  No …...…………………………… 2 |  |
| 221 | Did you find a change in your daily routine after your marriage? | Yes ……………………………….. 1  No …...…………………………… 2 |  |
| 222 | Did you lose your interest in socialising with people after your marriage? | Yes ……………………………….. 1  No …...…………………………… 2 |  |
| 223 | Did you stop going out frequently after your marriage? | Yes ……………………………….. 1  No …...…………………………… 2 |  |
