# Supplementary material for: Early marriage and marital satisfaction among young married men in rural Uttar Pradesh, India
Source: BMC Res Notes. 2023 Jan 27;16:6. doi: 10.1186/s13104-023-06271-9 (PMC9881292; doi:10.1186/s13104-023-06271-9)
Supplement: Supplementary file 4 — Additional file 4. Interview schedule for father. [file 13104_2023_6271_MOESM4_ESM.docx]

**Child Grooms: A Study of Early Marriage and its Repercussions on Young Men in Rural Uttar Pradesh**

SCHEDULE NO:

CONFIDENTIAL

For Research Purpose Only


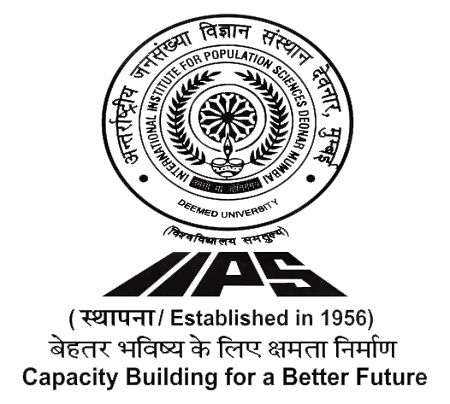


**Interview Schedule for Father**

| **IDENTIFICATION** | |
| --- | --- |
| DISTRICT: | VILLAGE: |
| TEHSIL: | NAME OF RESPONDENT: |
| DATE: |  |

| **Interview Status** | |
| --- | --- |
| Completed ………… 1 | Not at Home ………………. 3 |
| Incomplete ………… 2 | Refused ……………………. 4 |

**SECTION I: HOUSEHOLD INFORMATION**

I would like to have some information about the people who usually live in your household

| LINE NO  001 | USUAL RESIDENTS OF THE HH | RELATIONSHIP WITH THE HEAD OF HH | SEX | AGE | MARITAL STATUS | EDUCATION  (if age >5 years) | WORKING STATUS | | |
| --- | --- | --- | --- | --- | --- | --- | --- | --- | --- |
|  | Please tell me the names of the persons who usually live in your HH starting with the head of the HH | What is the relationship of (Name) to the head of the household? | Is (Name) male or female?  01= Male  02= Female  03= Others | How old is (Name)?  (In Completed Years) | What is the current marital status of (Name)? | What is the highest standard (Name) has completed? | What is the current working status of (Name)?  1= Working  2= Not Working | Is (Name) in full time or part time employment (If working)  1 = Yes  2 = No | What is the nature of employment  (if working) |
| (1) | (2) | (3) | (4) | (5) | (6) | (7) | (8) | (9) | (10) |
| 01 |  |  |  |  |  |  |  |  |  |
| 02 |  |  |  |  |  |  |  |  |  |
| 03 |  |  |  |  |  |  |  |  |  |
| 04 |  |  |  |  |  |  |  |  |  |
| 05 |  |  |  |  |  |  |  |  |  |
| 06 |  |  |  |  |  |  |  |  |  |
| 07 |  |  |  |  |  |  |  |  |  |
| 08 |  |  |  |  |  |  |  |  |  |
| 09 |  |  |  |  |  |  |  |  |  |
| 10 |  |  |  |  |  |  |  |  |  |
| 11 |  |  |  |  |  |  |  |  |  |
| 12 |  |  |  |  |  |  |  |  |  |
| 13 |  |  |  |  |  |  |  |  |  |
| 14 |  |  |  |  |  |  |  |  |  |
| 15 |  |  |  |  |  |  |  |  |  |
| 16 |  |  |  |  |  |  |  |  |  |
| 17 |  |  |  |  |  |  |  |  |  |

Please add sheets and columns if more members are in the household.

| Codes for Q. 3 | Codes for Q. 6 | Codes for Q. 7 | Codes for Q. 10 |
| --- | --- | --- | --- |
| 01= Head | 01= Currently Married | 01= No education  02= No education, but can read and write | 01 = Government Sector |
| 02= Husband or wife | 02= Widowed | 03= Primary | 02 = Private Sector |
| 03= Son or Daughter | 03= Divorced | 04= Secondary | 03 = Self-employed/ Business |
| 04= Son-in-law or Daughter-in-law | 04= Separated | 05= Higher Secondary | 04 = Working on own land |
| 05= Grand Child | 05= Never Married | 06= Graduation | 05 = working on Other’s land |
| 06= Brother or Sister |  | 07= Post Graduation | 96 = Others (Specify) |
| 07= Niece/ Nephew |  | 08= Professional |  |
| 08= Other Relatives |  |  |  |
|  |  |  |  |
|  |  |  |  |

| S. NO. | Questions | Coding categories | Skip/ Go to |
| --- | --- | --- | --- |
| 11 | What is the religion of the head of the household | Hindu………………………..…. 1  Muslim………….......................... 2  Other (Specify)……….................. 96  Don’t Know ….………................. 98 |  |
| 12 | What is the Caste of the head of the household | Scheduled caste …... ……………… 1  Scheduled Tribe …........................... 2  Other Backward Caste …….……… 3  Other (Specify) ………………….. 96  Don’t Know ……………………... 98 |  |
| 13 | What is the language you generally speak at home | Hindi ……………………………... 1  English …………………………… 2  Urdu …………. ………………….. 3  Other (Specify) …………………. 96 |  |
| 14 | Have you always lived in this village | Yes ………………………...…....... 1  No ………………….. ………........ 2 | Skip to Q. 16 |
| 15 | How long have you been living in this village | Months ……………………………  Years ……….……………………...  Don’t Know ……...………………98 |  |
| 16 | Note the type of house (Observe and Record) | Kuccha House ……………...…….. 1  Semi Pucca house ...….……..…….. 2  Pucca House ………………..…….. 3 |  |
| 17 | How many rooms are there in your home excluding bathrooms, balconies, or hallways but including kitchen? | Number of rooms ….. |  |
| 18 | Of these rooms, how many are used for sleeping? | Number of rooms ….. |  |
| 19 | Do you have separate room for kitchen? | Yes …………………...…………... 1  No ………………………………... 2 |  |
| 20 | What is the main source of drinking water for the household? | Piped water ………………..……... 1  Water from spring ………………... 2  Rain water ………………..…......... 3  Tanker ………………………......... 4  Bottled water/ purchased water …... 5  Hand pump ……….………………. 6  Tube well/ bore well ……………... 7  Other (Specify) ………………... 96 |  |
| 21 | Does your household have | Yes No  Electricity ……….…………… 1 2  A cot or bed …….……………. 1 2  A table ………….……………. 1 2  An electric fan ….……………. 1 2  Radio …………….…………... 1 2  Black & white TV .………….. 1 2  Colour TV ……….…………... 1 2  Sewing Machine ….…………. 1 2  Telephone ………….………… 1 2  Mobile ……………………… 1 2  Computer/ Laptop ….………… 1 2  Refrigerator ………………….. 1 2  AC/ Cooler …………………… 1 2  Washing Machine ……………. 1 2  Bicycle ………………………. 1 2  Motor Cycle ………………….. 1 2  Car …………………………… 1 2  Tractor ……………………...… 1 2 |  |
| 22 | What type of fuel does HH mainly use for cooking | Electricity ……………………….. 01  LPG/ Natural Gas ………………. 02  Biogas …………………………... 03  Kerosene ………………………... 04  Coal ……………………………... 05  Wood …………………………… 06  Agriculture Waste …….………... 07  Dung cakes ……………………… 08  Others (Specify) ………………… 96 |  |
| 23 | Does this household have latrine? | Yes …………………...…………... 1  No ………………………………... 2 | If No, Skip to 23 B |
| 23. A | Who has built this toilet | Respondent himself ……………….. 1  Government through SWM ……….. 2  Govt. through other schemes ……… 3 |  |
| 23. B | If No, Where HH members go for latrine? | Use open space …………….……... 1  Share neighbour’s latrine …………. 2  Public latrines …………………….. 3 | Skip to Q. 24 |
| 23. C | If Yes, What type of toilet facility do members of your household use? | Flush Toilet ……………….……… 1  Pit latrine with slab ……….……… 2  Pit latrine without slab …….……... 3  All members use open space ……… 4  Male use open space ………………. 5  Other (Specify) …………….…… 96 |  |
| 24 | Does any member of this household own this house | Yes …………………...…………... 1  No ………………………………... 2 | Skip to Q 26 |
| 25 | Who owns this house (Write S. No. from Q. 1 in household roaster) | S. No …………………………. ( ) |  |
| 26 | Does your household have any agricultural land? | Yes …………………...…………... 1  No ………………………………... 2 | Skip to Q 29 |
| 27 | How much? (acres/ beegha, mention) |  |  |
| 28 | Who owns this land (Write S. No. from Q. 101 in household roaster) | S. No. …………………………( ) |  |
| 29 | What is the main source of lighting in your house? | Electricity ………………………… 1  Kerosene ………………………..... 2  Gas ……………………………….. 3  Solar energy ……………………... 4  Others (specify) …………………. 96 |  |
| 30 | Does your household have any of the following? | \| Sr. No. \| Animals \| Yes/No \| Number \| \| --- \| --- \| --- \| --- \| \| 1 \| Cow \|  \|  \| \| 2 \| Buffalo \|  \|  \| \| 3 \| Goat \|  \|  \| \| 4 \| Sheep \|  \|  \| \| 5 \| Bulls \|  \|  \| \| 6 \| Others \|  \|  \| |  |
| 31 | Does your household has ration card? | Yes …………………...…………... 1  No ………………………………... 2 | Skip to Q 33 |
| 32 | Colour of ration card | Yes …. 1 No …. 2  Yellow (BPL) …….…………. 1 2  Yellow (Antyodaya scheme) …1 2  Saffron (APL; Patr Grahasthi) 1 2  Others Specify ………………… 96 |  |
| 33 | What is the total monthly income of your household? (In Rs.) | Below 5000 …….………………... 1  5001 – 10000 …….………………. 2  10001 – 20000 ….………………... 3  20001 – 50000 ….………………... 4  50001- 100000 ….…………………5  Above 1 Lakh …….……………….. 6 |  |

**SECTION II: PERSONAL INFORMATION:**

| S. No. | Questions | Coding Categories | Skip/ Go to |
| --- | --- | --- | --- |
| 34 | How old were you on your last birthday? | Age in completed years |  |
| 35 | What is your current marital status? | Married …………………………… 1  Separated …………………………. 2  Widowed …………………………. 3  Divorced ….………………………. 4 |  |
| 36 | What is the age of your spouse? (if currently married and alive) | \|  \| \| --- \|   Don’t know …………………… 98 |  |
| 37 | What is her level of education? | No education ……………………... 1  Literate but, no formal education . 2  Primary …………………………… 3  Secondary ………………………… 4  Higher secondary …………….……5  Graduation ………………………... 6  Above graduation ………………… 7  Don’t know …………………….. 98 |  |
| 38 | What is the age difference between you and your wife? | \| (Years) \| \| --- \|   Don’t know ……………………. 98 |  |
| 39 | At what age you were married? | \|  \| \| --- \|   Don’t know …………………….. 98 |  |
| 40 | What was the age of your wife at the time of your marriage? | \|  \| \| --- \|   Don’t know ……………………. 98 |  |
| 41 | Can you read and write? | Able to read only …………………. 1  Able to write only ………………... 2  Able to read and write ……………. 3  Cannot read or write ……………… 4 |  |
| 42 | What is the highest level of education that you have completed? | No education ……………………... 1  Literate but no formal education … 2  Primary …………………………… 3  Secondary ………………………… 4  Higher secondary ………………… 5  Graduation ……………………….. 6  Above graduation ………………… 7  Don’t know ……………………... 98 |  |
| 43 | Years of schooling? | 0 to 30 years |  |
| 44 | What is your current working status? | Currently working ………………... 1  Re-employed after retirement …… 2  Retired ………................................ 3  Home- maker …………………….. 4  Working on own agricultural land 5  Working on others agricultural land 6  Not working ………........................ 7  Marginal work ……………………. 8  Any Other (Specify) …………… 96 |  |
| 45 | Primary Source of income | From agriculture …………………. 1  From daily work ……………….. 2  From govt. job …………………… 3  From private job …………………. 4  From shop/petty trade …………… 5  Business ………………………….. 6  Others (specify) …………………. 96 |  |
| 46 | Your monthly income (Average) | In Rupee |  |
| 47 | Do you read a newspaper or magazine? | Almost every day ………………… 1  At least once a week ……………... 2  Less than once a month …………..3  Not at all ………………………….. 4 |  |
| 48 | Do you listen to the radio? | Almost every day ………………… 1  At least once a week ……………... 2  Less than once a month …………..3  Not at all ………………………….. 4 |  |
| 49 | Do you watch television? | Almost every day ………………… 1  At least once a week ……………... 2  Less than once a month ………….3  Not at all ………………………….. 4 |  |
| 50 | How many children do you have? |  |  |
| 51 | Of all the children, number of sons and daughters | Sons  Daughters |  |
| 52 | How many of your children are married? | Sons  Daughters |  |
| 53 | At what age each of your children got married and the age of their spouse at the time of marriage?  (eldest to youngest) | \| **S. No.** \| **Sex (M/F)** \| **Age at marriage** \| **Age at marriage (Spouse)** \| \| --- \| --- \| --- \| --- \| \| 1 \|  \|  \|  \| \| 2 \|  \|  \|  \| \| 3 \|  \|  \|  \| \| 4 \|  \|  \|  \| \| 5 \|  \|  \|  \| \| 6 \|  \|  \|  \| \| 7 \|  \|  \|  \| |  |
| 54 | To what extent did your son/s complete their education at the time of marriage?   \| Son \| 1st \| 2nd \| 3rd \| 4th \| 5th \| \| --- \| --- \| --- \| --- \| --- \| --- \| \| No education \|  \|  \|  \|  \|  \| \| Primary \|  \|  \|  \|  \|  \| \| Secondary \|  \|  \|  \|  \|  \| \| Higher secondary \|  \|  \|  \|  \|  \| \| Graduation \|  \|  \|  \|  \|  \| \| Post-graduation \|  \|  \|  \|  \|  \| \|  \|  \|  \|  \|  \|  \| | |  |
| 55 | To what extent did your daughter/s complete their education at the time of marriage?   \| Daughter \| 1st \| 2nd \| 3rd \| 4th \| 5th \| \| --- \| --- \| --- \| --- \| --- \| --- \| \| No education \|  \|  \|  \|  \|  \| \| Primary \|  \|  \|  \|  \|  \| \| Secondary \|  \|  \|  \|  \|  \| \| Higher secondary \|  \|  \|  \|  \|  \| \| Graduation \|  \|  \|  \|  \|  \| \| Post-graduation \|  \|  \|  \|  \|  \| \|  \|  \|  \|  \|  \|  \| | |  |

**SECTION III: MARRIAGE PRACTICES AND NORMS**

Now, I would like to ask you some questions about marriage customs in your community, for a family like yours

| S. No. | Questions | Coding Categories | Skip/ Go to |
| --- | --- | --- | --- |
| 56 | If both boys and girls are in the marriageable ages, who will get marry first | Boy ……………………………... 1  Girl ……………………………... 2 |  |
| 57 | Are all your children married within your own community | Yes …………………………….. 1  No ……………………………... 2 |  |
| 58 | Do you think it is alright to marry outside caste/ community | Yes ……………………………... 1  No ……………………………… 2 |  |
| 59 | Do you or other people in your household asked for their (children) interest and decision in marriage | Yes ……………………………... 1  No ……………………………… 2  Sometimes ……………………… 3  Don’t know …………………….. 4 |  |
| 60 | In your household, who all are involved in the decision making process of the marriages of your children? | Respondent alone ………………. 1  Respondent with spouse ……….. 2  Person to be married …………… 3  Person to be married along with the other family members …………. 4  Respondent along with other family members ……………………….. 5 |  |
| 61 | How did you decide that your son is ready for marriage? | He is 14 years and above ………. 1  He is 18 years and above ………. 2  He can earn his own living …….. 3  He has stopped studying ……….. 4  people started asking about his marriage …………….…..……… 5  He wants to get married ………... 6  Threatened that he may run away and marry someone from outside caste ……………………………. 7  Not marrying him can lead to indulge in unsafe or outside marriage sexual behaviour …….. 8  Anytime a good proposal comes 9  Whenever it is easy to arrange  money for his marriage (economic reasons) ………………………. 10  Any other reason (specify) …… 96 |  |
| 62 | How did you decide that your daughter is ready for marriage? | She is 10 years and above ……… 1  She is 15 years and above …….. . 2  She is 18 years and above ……… 3  She has attained puberty …….. ... 4  She has completed her education 5  She has stopped studying ………. 6  People started asking about her marriage ………………...……… 7  She wants to get married ……….. 8  Threatened that she may run away and marry someone from outside her caste ………………………... 9  Boys started eve teasing her ….. 10  Anytime a good proposal comes 11  Whenever it is easy to arrange money for her marriage (economic reasons) ………………………. 12  She got into an affair …………. 13  For her own safety as everyone go out for work and she stay alone 14  Any other reason (specify) ……. 15 |  |
| 63 | Generally in your family, do the groom/bride saw the partner before marriage? | Yes ……………………………... 1  No ……………………………… 2 |  |
| 64 | Did they interact with the bride/groom before marriage? | Yes ……………………………... 1  No ……………………………... 2 |  |
| 65 | Do you think it is better to marry son in early ages? | Yes …………………………….. 1  No ……………………………… 2 |  |
| 66 | Do you think it is better to marry daughter in early ages? | Yes ……………………………... 1  No ……………………………… 2 |  |
| 67 | Do you think it is right or appropriate to marry children early? | Yes ……………………………... 1  No ……………………………… 2 |  |
| 68 | Do other people in your community/ caste do the same? | Yes ……………………………... 1  No ……………………………… 2 |  |
| 69 | According to you, what is the ideal age gap between husband and wife? | Equal In age ……………………. 1  0-2 years ………………………... 2  3-5 years ………………………... 3  6-8 years ………………………... 4  9-10 years ………………………. 5  10+ years ……………………….. 6 |  |
| 70 | Will you agree for the marriage of your son to a girl with equal in age? | Yes …………………………….. 1  No ………………………………. 2 |  |
|  | If no, what are the reasons? | Yes ……… 1 No ………….. 2  Against the custom ………... 1 2  She will dominate …………. 1 2  Difficult to control her …….. 1 2  Will reduce the dowry ……... 1 2  Any Other (Specify) ………….. 96 |  |
| 71 | Will you agree for the marriage of your son to a girl who is older than him? | Yes …………………………….. 1  No ………………………………. 2 |  |
| 72 | If No, What are the reasons? | Yes ……… 1 No ………….. 2  Against the custom ………... 1 2  He may feel inferiority complex 1 2  She will dominate …………. 1 2  Difficult to control her …….. 1 2  Will reduce the dowry ……... 1 2  Any Other (Specify) ………….. 96 |  |
| 73 | What are the characteristics do you think an appropriate bride should have? (Multiple responses are allowed, but to be marked in the increasing order of preference using serial number)  She should be educated  She should be earning/ ready to work outside home/ agriculture  She should be beautiful  She should know how to do all the household work  She should be from good family  She should be adjusting with all the family members  She should not have any affair before marriage  She should be virgin  Any other (specify) | |  |

**SECTION IV: COST OF MARRIAGE**

| S. No. | Questions | Coding Categories | Skip/ Go to |
| --- | --- | --- | --- |
| 74 | Do you think arranging marriage is an expensive affair? | Yes ……………………………... 1  No ……………………………… 2 |  |
| 75 | Cost of marriage is higher when | Son is getting married …………. 1  Daughter is getting married ……. 2 |  |
| 76 | How much did you spent on each of your son’s marriage? | \| No. \| Name of the son (elder first) \| Approx.. money spent (Rs.) \| \| --- \| --- \| --- \| \| 1 \|  \|  \| \| 2 \|  \|  \| \| 3 \|  \|  \| \| 4 \|  \|  \| \| 5 \|  \|  \| |  |
| 77 | How much did you spent on each of your daughter’s marriage? | \| No. \| Name of the daughter (elder first) \| Approx. money spent  (Rs.) \| \| --- \| --- \| --- \| \| 1 \|  \|  \| \| 2 \|  \|  \| \| 3 \|  \|  \| \| 4 \|  \|  \| \| 5 \|  \|  \| |  |
| 78 | How much did you spent on marriage of your last son? | Amount in Rs. |  |
| 79 | How much did you spent on marriage of your last daughter? (Including Dowry) | Amount in Rs. |  |
| 80 | What were the major components on which the expenses were incurred during marriage of your son? | Yes …….. 1 No ………2  Food ………………………... 1 2  Decoration …………………. 1 2  Gifts ………………………... 1 2  Clothes for relatives and bride 1 2  Guest living arrangements …. 1 2  Transport …………………... 1 2  Music (band) ………………...1 2  Jewellery …………………….1 2  Others (specify) ………………. 96 |  |
| 80 | What were the major components on which the expenses were incurred during marriage of your daughter? | Yes …….. 1 No ………2  Food ………………………... 1 2  Decoration …………………. 1 2  Gifts ………………………... 1 2  Clothes for relatives and groom 1 2  Guest living arrangements …. 1 2  Transport …………………... 1 2  Music (band) ………………...1 2  Jewellery …………………….1 2  Dowry …………………… 1 2  Others (specify) ………………. 96 |  |
| 81 | How many guests did you invite for the marriage ceremony/ | Below 50 ………………………. 1  51-100 ………………………….. 2  101-200 ………………………… 3  201-300 ………………………… 4  301-400 ………………………… 5  401-500 ………………………… 6  More than 500 ………………….. 7 |  |
| 82 | What kind of gifts did you give to guests? | Yes ……. 1 No ……..2  Gold/silver coins ................ 1 2  Utensils …………………... 1 2  Clothes …………………… 1 2  Jewellery …………………. 1 2  Cash (amount) …………… 1 2  Others (specify) ……………… 96  Nothing ……………………… 98 |  |
| 83 | What were the expenses incurred in different arrangements for the marriage? (approx.) | \| No. \| Things \| Approx. money spent \| \| --- \| --- \| --- \| \| 1 \| Functions / Ceremony \|  \| \| 2 \| Place/ Hall \|  \| \| 3 \| Food / Feast \|  \| \| 4 \| Gifts \|  \| \| 5 \| Clothes for relatives/ bride \|  \| \| 6 \| Guest living arrangements \|  \| \| 7 \| Transport \|  \| \| 8 \| Music (band) \|  \| \| 9 \| Jewellery \|  \| \| 10 \| others \|  \| |  |
| 84 | Did you receive any gifts from your in-laws at the time of the marriage of your son? | Yes ……………………………... 1  No ……………………………… 2 | Skip to  Q. 87 |
| 85 | What were those gifts? | Yes ………. 1 No ….. 2  Clothes ………………… 1 2  Sweets …………………. 1 2  Jewellery ………………. 1 2  Gold/silver coin ……….. 1 2  Utensils ………………... 1 2  Television ……………... 1 2  Refrigerator …………… 1 2  Washing machine ……… 1 2  Fan …………………….. 1 2  Mobile ………………… 1 2  Bike (two wheeler) …… 1 2  Sofa ……………………. 1 2  Bed ……………………..1 2  Car (four wheeler) ……... 1 2  Computer/ laptop ………. 1 2  Land ……………………. 1 2  House ………………… 1 2  Other (specify) ……………... 96 |  |
| 86 | Do you think they gave enough gifts in the marriage? | Yes …………………………….. 1  No ……………………………… 2 |  |
| 87 | Did you ask for any particular gifts/ commodities? | Yes …………………………….. 1  No ……………………………… 2 | Skip to Q. 89 |
| 88 | If yes, particularly what? | Yes ………… 1 No …….. 2  Gold jewellery …………. 1 2  Car ……………………... 1 2  Bike ……………………. 1 2  Furniture ………………. 1 2  Refrigerator ……………. 1 2  Washing Machine ……… 1 2  Land ……………………. 1 2  House ………………… 1 2  Others (specify) ………………. 96 |  |
| 89 | Did you receive any money as a token of gift? | Yes …………………………….. 1  No ……………………………… 2 | Skip to  Q. 91 |
| 90 | How much money you received as gift? | In Rupee ( ) |  |
| 91 | Do you think parents should give something to their daughters during marriage? | Yes …………………………….. 1  No ……………………………… 2 | Skip to  Q. 93 |
| 92 | If yes, why you think so? | Yes ………… 1 No …….. 2  Out of love ………………... 1 2  That is a custom …………... 1 2  She is dependent ………….. 1 2  It is her share in parental property ……………………………. 1 2  Manifestation of status ……. 1 2  Other (specify) ………………. 96 |  |
| 93 | Why do you think it is important to get dowry? | Yes …. 1 No ………. 2  It is bride’s share in parental property …………………… 1 2  For equality of status and manifestation of status …….. 1 2  To follow tradition ………… 1 2  To compensate expense incurred for educating/job of the groom… 1 2  To get back what was lost due to marrying off daughter ……… 1 2  To set up a life of their own …1 2  Other (specify) ………………... 96 |  |
| 94 | Generally how it is spent? | Yes ……. 1 No ……….. 2  Invest in business/ agriculture ………………………………. 1 2  Invest in land, house or other properties ……….................. 1 2  Pay back loan ……………... 1 2  Marrying off other siblings … 1 2  Security for job ……............. 1 2  Facilitate future study of groom ……………………………… 1 2  To fund the studies of other siblings …………………………….. 1 2  Compensate for the marriage expenditure ………………… 1 2  Saved for future expenses for bride and groom ………………… 1 2  Others (specify) ………………. 96 |  |
| 95 | Why did you demand dowry? | Yes ………. 1 No ……… 2  It is customary …………….. 1 2  I also paid for my daughter’s marriage …………………… 1 2  My son is qualified ………… 1 2  My son has a very good job …1 2  Now their daughter is our liability ……………………………… 1 2  Its bride’s share in parental property ……………………………… 1 2  I did not want, but my family members demanded ………... 1 2  Other reasons (specify) ……. 96  Not applicable (did not take dowry) ………………………………... 98 |  |
| 96 | Do your relatives and friends in your community also have dowry practise? | Yes …………………………….. 1  No ……………………………… 2 |  |
| 97 | What generally determines the amount of dowry? | Yes ………. 1 No ……….. 2  Boy’s educational qualification ……………………………… 1 2  Boy’s occupation/ current job 1 2  Caste …………………………1 2  Girl’s age …………………… 1 2  Girl’s educational qualifications ……………………………….1 2  Girl’s appearance …..………. 1 2  Status of boy’s family ………. 1 2  Status of girl’s family ………. 1 2  Demand of the groom’s family 1 2  Societal pressure ……………. 1 2  Other reason (Specify) ………... 96 |  |
| 98 | Do you think it is correct/ rightful to accept dowry? | Yes …………………………….. 1  No ……………………………… 2 |  |
| 99 | Have you heard about the prevention of dowry act? | Yes …………………………….. 1  No ……………………………… 2 |  |
| 100 | Would you marry your son from a family where they refuse to give dowry? | Yes …………………………….. 1  No ……………………………… 2 |  |
| 101 | Do age at marriage of boys has anything to do with the amount of dowry they receive? | Yes …………………………….. 1  No ……………………………… 2 |  |
| 101. A | When dowry is higher | When groom is older than bride 1  When groom is younger than bride …………………………………. 2 |  |

**SECTION V: REGARDING LATEST MARRIAGE DONE IN THE HOUSEHOLD/ REASONS FOR EALRY MARRIAGE**

| S. No. | Questions | Coding Categories | Skip/ Go to |
| --- | --- | --- | --- |
| 102 | How many marriage proposals were rejected before finalizing the marriage of your son? | Selected the first proposal ….…… 1  1-3 proposals ……………………. 2  4-5 proposals …………………… 3  More than 5 proposals ………….. 4 | Skip to Q. 104 |
| 103 | What was the most important reason for rejecting the proposals? | Son didn’t liked the bride ………. 1  Family was not good …………… 2  Family not ready to fulfil the dowry demands .………………………. 3  Caste Issues …………………….. 4  The girl was working (Job) …….. 5  Number of siblings in the girl’s family ……………………………. 6  Economic status of the girl’s family …………………………………. 7  Any other (specify) …………… 96 |  |
| 104 | What was the age of your son at the time of his marriage? |  |  |
| 105 | What was the age of your daughter-in-law at the time of her marriage? |  |  |
| 106 | In your community, are the marriages decided within the nearby villages? | Yes …………………………….. 1  No ……………………………… 2 |  |
| 107 | What is the distance of your daughter-in-law’s home from here | ( ) in kms. Approx.. |  |
| 108 | Was any middleman/broker involved in arranging your son’s marriage? | Yes …………………………….. 1  No ……………………………… 2 |  |
| 109 | Numbers of sisters and brothers unmarried at the time of his marriage? | Brothers  Sisters |  |
| 110 | Why did you decide that he should get married now? | Yes …… 1 No ………. 2  Part of culture …………….. 1 2  Fear of son marrying outside caste …………………………….. 1 2  Economic needs of the family1 2  Social pressure ……………. 1 2  It is difficult to get good bride later …........................................... 1 2  Need for a helping hand at home …………………………….. 1 2  Son is jobless/uneducated/ not studying ………………..… 1 2  Arrival of good proposal …. 1 2  Need to arrange money for daughter’s marriage ……… 1 2  Many other sons and daughters to marry ………………………. 1 2  Other reason (specify) ………… 96 |  |
| 111 | Was he studying at the time of marriage? | Yes …………………………….. 1  No ……………………………… 2 | Skip to Q. 114 |
| 112 | Did he stop going school/ college after marriage? | Yes …………………………….. 1  No ……………………………… 2 |  |
| 113 | When did he discontinue his education? | When he completed class 5 …….. 1  When he completed class 8 …….. 2  When he completed class 10 …… 3  When he completed class 12 ….. 4  When marriage got fixed …….. 5  When he got a job …………….. 6  When he got married ………….. 7  He is uneducated ……………… 8  Any other (specify) …….………96 |  |
| 114 | Why he discontinue his education? | He failed in class/ not interested in studies ..……………………….. 1  Could not afford it further …….. 2  School was far …………………. 3  He has to take care of younger siblings at home ………………… 4  His marriage was fixed ………… 5  He got a job …………………….. 6  He is uneducated ……………….. 7  Other reasons ………………… 96 |  |
| 115 | What was his age when he left the school? | Age ………… |  |
| 116 | Did he want to study further? | Yes …………………………….. 1  No ……………………………… 2 |  |
| 117 | Was he happy with his marriage being fixed? | Yes …………………………….. 1  No ……………………………… 2 |  |
| 118 | Did you take his consent before fixing the marriage? | Yes …………………………….. 1  No ……………………………… 2 |  |
| 119 | Did you allow him to meet his spouse before marriage? | Yes …………………………….. 1  No ……………………………… 2 |  |
| 120 | Did he want to get marry at that time? | Yes …………………………….. 1  No ……………………………… 2 |  |
| 121 | Did he refused to marry the person you have chosen? | Yes …………………………….. 1  No ……………………………… 2 | Skip to Q. 123 |
| 122 | If yes, how did you convince him? | Through persuasion …………….. 1  Threatening …………………….. 2  Asked somebody to convince him 3  Did not care to convince him…… 4  Others (specify) ……………….. 96 |  |
| 123 | Is marrying a boy early is a common practice in your community? | Yes …………………………….. 1  No ……………………………… 2 |  |
| 124 | Do you think allowing a son to study higher leads to complications for marriage? | Yes …………………………….. 1  No ……………………………… 2 | Skip to Q. 126 |
| 125 | If yes, what kind of complications? | He may become modern ……….. 1  He may be more demanding …… 2  He may want to move outside home ………………………………….. 3  He may decide to marry on his own ………………………………….. 4  He may develop aspirations to study further ……………………. 5  Others (specify) ………………. 96 |  |
| 126 | Do you know about the legal age at marriage of boys and girls? | Yes …………………………….. 1  No ……………………………… 2 | Skip to Q. 128 |
| 127 | What is the legal age of marriage in India? | Boys  Girls |  |
| 128 | Why did you not follow the minimum age at marriage while marrying your son? | I don’t agree with the law ……… 1  I agree with the law, but due to social customs/ Practices………… 2  I agree with the law, but due to economic conditions …………… 3  I agree with the law, but received a good proposal ………………….. 4  My son want to get married …… 5  Girl’s side agreed to my demands 6  Any other (specify) …………… 96 |  |
| 129 | Do you think it is important to have a minimum age at marriage for boys? | Yes …………………………….. 1  No ……………………………… 2 |  |
| 130 | Are the marriages of your children registered? (at least last marriage) | Yes …………………………….. 1  No ……………………………… 2 | Skip to Q. 132 |
| 131 | Where did you registered it? | Nagar Nigam …………………… 1  Nagar Palika ……………………. 2  Gram Panchayat Office ………… 3  Marriage registration office ……. 4  Court …………………………… 5  Not registered ………………….. 6 |  |
| 132 | Do other families in your village register the marriages? | Not at all ……………………….. 1  Sometimes ……………………… 2  Each time ………………………. 3  Not sure ………………………… 4 |  |
| 133 | What do you think can help in reducing the incidences of early marriage of boys? | By educating boys ………..……. 1  Punish those who get married before the legal age …..………… 2  Cash incentives for boys ……….. 3  Through awareness programs ….. 4  By targeting poverty …………… 5  Any other (specify) …………… 96 |  |
| 134 | What can be done to reduce early marriage of girls? | Avoid early marriage of boys ….. 1  By educating girls ……………… 2  Punish those who get married before the legal age …………….. 3  Cash incentives for girls ……….. 4  Through awareness programs ….. 5  By targeting poverty …………… 6  Any Other (Specify) …………… 96 |  |
| 135 | Why did you chose to marry your son early? | He was not good at studies …… 1  He himself want to get married … 2  He had the love affair ….……….. 3  Feared that he may run away with another girl ………………….….. 4  He dropped out of school ………. 5  He was in bad company …...…… 6  Need someone at home to look after the household work …………….. 7  Offered a good dowry ………….. 8  Was in need of money …………...9  Other sons and daughters to be married off after him ………….. 10 |  |
